# Annotations

**Published:** 1906-06-09

**Authors:** 


### ANNOTATIONS.

### Reform in Medical Study.

The medical curriculum finds a never-failing supply of critics. No sooner has one school of opinion secured effective recognition of its views than agitation again arises either for still further developments or for a return to former educational plans. The existing arrangements in this respect are not more fortunate than their predecessors, and the present generations of reformers are as confident of the value of their several proposals as were those of an earlier day. Among such proposals one of the most recent is that the course of medical education should recognise the necessity of distinguishing between the students who intend to become practical physicians and those who aspire to the work of the laboratory or the desk of the professor. It is argued that a fundamental distinction exists between these groups. The practical physician, it is urged, is concerned with the cure of the individual patient, and must be trained at the bedside as a clinical practitioner. The scientist and teacher, on the other hand, find their work in the advance of medical science and in the communication of this advance to the next generation, and for them, therefore, in their embryonic stage, hospital practice is relatively unimportant, and laboratory work and pure science have predominating claims. All this is very plausible, and contains a measure of truth. But it is essentially vicious in respect to the fact that it is an endeavour to apply a sound principle prematurely, and to recognise distinctions before they are really manifest. How, in the early stages of a student's career is it possible to anticipate the lines of his future development? It is only at a much later stage, in the great majority of cases, that personal choice and capacity show their peculiar and individual bent. That a certain proportion of medical practitioners will continue to turn towards laboratory rather than clinical practice is doubtless true. But it must never be forgotten that such practice, to be of value, must associate itself with clinical facts and clinical methods, and that the highest interests of medicine demand that the laboratory worker, equally with the practical physician, must have enjoyed the discipline of thorough hospital training. A division of labour will surely manifest itself in time. But to attempt to establish this in advance of the facts can only prove disastrous.

### The Kinematograph in Medicine.

The demonstration recently given by Dr. Walter G. Chase to the Edinburgh Medico-Chirurgical Society emphasised very emphatically how valuable as aids to teaching may be some of the newer photographic developments. The possibilities of the kinematograph in this direction have doubtless occurred to others, but no one seems to have translated them into action with the fulness and success attained by Dr. Chase. His films included practically the whole range of those abnormal movements which are recognised as evidences of disease, not excepting even the relatively elaborate phenomena of an epileptic fit. The thoroughness with which the subject has been pursued may be judged from the statement that the films exhibiting epileptic seizures measure 1,500 feet and contain 22,500 minute pictures of attitudes assumed during the convulsions. The value of such records for purposes of teaching clinical medicine and surgery can hardly be exaggerated. In the absence of illustrative cases—and to command a ready supply of these is not possible—the student is reduced to that most tiresome of all methods, the study and acquisition of verbal descriptions. The weariness of this, and its practical failure, are known to every teacher and examiner. Hence Dr. Chase's anticipation that a kinematograph outfit will be recognised as a necessary part of a regular teaching equipment is both reasonable and inevitable. The modern student has many competing schools offered to his choice, and up-to-date educational methods will sooner or later determine the issue. In this column, on a former occasion, we have urged the value of the clinical picture gallery, and the claims of the kinematograph seem to be equally well founded.

### Sweated Industries.

The Sweated Industries Exhibition at present in progress at Queen's Hall under the auspices of the *Daily News* brings into prominence a peculiarly pathetic aspect of crowded urban life. That in every large industrial community there are thousands working long hours under dreadful conditions and for totally inadequate remuneration there can be no question, and our contemporary will gain sympathy from all fair-minded people, regardless of political creed, for its plucky attempt to ventilate the subject and enlist public interest. In a publication explanatory of the exhibition are many articles dealing with the different phases of the problem as affecting different industries. Amongst these is one entitled "Suggested Remedies." In it the particular evils attendant upon home labour—which is *par excellence* that which is most sweated—are referred to the four following causes: 1. Excessive hours. 2. Unsuitability of work-place. 3. Employment of child labour. 4. Low pay. It is sufficiently obvious that, of these four, the first three are little more than incidental consequences of the last. Where remuneration is inadequate there long hours and the enlistment of all possible help become inevitable. How, then, is it that people are to be found who are willing to undertake laborious days upon such terms? We are told, and no doubt with justice, that this condition of affairs is the fruit of competition, and that the remedy is co-operation as opposed to competition. Here we touch upon matters of economic opinion, and many, no doubt, will refuse to subscribe to so large a rule. But all are at one upon the facts, and most, we suspect, will deprecate unrestricted competition. By this we mean the competition of those already partially provided for (as, for instance, by the earnings of husbands or brothers), with those to whom the same work is a matter of food and housing. The establishment of a standard minimum wage would obviate most of the attendant objections of the current system, and might be expected to appeal, without distinction of party, to all classes of the community.

